# Exploring *Rattus praetor* (Rodentia, Muridae) as a possible species complex using geometric morphometrics on dental morphology

**DOI:** 10.1016/j.mambio.2018.04.002

**Published:** 2018-09

**Authors:** Ardern Hulme-Beaman, Thomas Cucchi, Allowen Evin, Jeremy B. Searle, Keith Dobney

**Affiliations:** a Department of Archaeology, Classics and Egyptology, University of Liverpool, 12-14 Abercromby Square, Liverpool L69 7WZ, UK; b Research Centre in Evolutionary Anthropology and Palaeoecology, School of Natural Sciences and Psychology, Liverpool John Moores University, Byrom Street, Liverpool L3 3AF, UK; c CNRS-Muséum National d'Histoire Naturelle, UMR 7209, Archéozoologie, Archéobotanique Sociétés, Pratiques et Environnement, 55 Rue Buffon, 75005 Paris, France; d Institut des Sciences de l'Evolution, Université de Montpellier, UMR CNRS, UM, EPHE, IRD 2 Place Eugène Bataillon, CC065, 34095 Montpellier, Cedex 5, France; e Department of Ecology and Evolutionary Biology, Cornell University, Corson Hall, Ithaca, NY 14853-2701, USA; f Department of Archaeology, Simon Fraser University, Burnaby, British Columbia, Canada

**Keywords:** Cryptic species, Phylogeography, Geometric morphometrics, Rat, Sahul

## Abstract

Taxonomic uncertainties in the *Rattus* genus persist due to among-species morphological conservatism coupled with within-species environmental variation in morphology. As a result, this genus contains a number of possible cryptic species. One important example can be found in *R. praetor*, where morphological studies indicate it is a possible species complex. Genetic studies of *R. praetor* (limited to analysis of mitochondrial DNA) have been inconclusive, but do indicate such subdivision. Here we use geometric morphometrics to explore this possible species complex by analysing the dental traits of 48 specimens from New Guinea and neighbouring regions. We find separate molar morphologies for Bougainsville Island, central New Guinea and west New Guinea which cannot be easily explained by different environmental factors (climate, precipitation and altitude), strongly suggesting the existence of a number of evolutionarily distinct taxa within what is currently called *R. praetor* thus supporting previous suggestions that *R. praetor* is a species complex. Our findings demonstrate the potential of advanced morphological analyses in identifying separate species, contrary to the claims of morphological conservatism. Future analyses should combine geometric morphometrics with genetic analyses over the species range and include sub-fossil specimens from the Bismarck archipelago and Solomon Islands to resolve the evolutionary history of *R. praetor*.

## Introduction

Small mammal genera are often speciose (Michaux et al., 2001), despite a high degree of morphological uniformity. Increasing numbers of cryptic species (i.e. two or more species erroneously classified as a single species) are now being identified with newly developed molecular techniques (Bickford et al., 2007; Paupério et al., 2012). However, although cryptic species by definition lack obvious differences in morphology, this does not mean that they are morphologically identical (e.g. Villemant et al., 2007). Geometric morphometrics represent a suite of powerful tools for examining morphological differences among groups (Adams et al., 2004), which have proven useful in the dectection of subtle morphological variation (Cucchi et al., 2006). Here we apply geometric morphometrics to a species of the genus *Rattus* which has previously been noted as likely including a number of cryptic species: *R. praetor* (Robins et al., 2014).

How to define species remains a hotly debated issue (Fišer et al., 2018) and the rapid increase in numbers of species defined solely by genetics is often regarded with scepticism (Carstens et al., 2013). The scepticism has become more apparent with increasing conflicts in species delimitations between mitochondrial and autosomal DNA datasets (e.g. *R. tanezumi* in Mainland Southeast Asia, Pagès et al., 2013). As a result, delimiting species should be based on multiple and diverse datasets including non-genetic characters (e.g. phenotypic, behavioural, ecological, etc.) (Fišer et al., 2018; Carstens et al., 2013). Here we take an approach to document the possible cryptic diversity of the previously proposed *R. praetor* species complex using exploratory geometric morphometric methods.

**Fig. 1:**
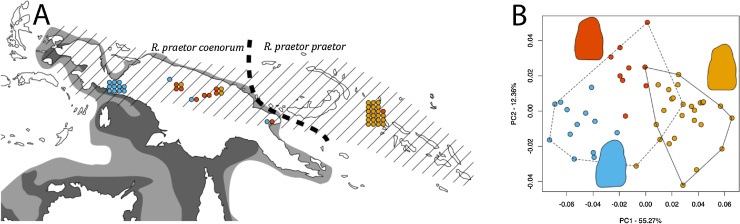
(A) Map of New Guinea and neighbouring islands indicating approximated distribution of *Rattus praetor* (cross hatching) (Taylor et al., 1982). All insular populations east and north of New Guinea belong to the subspecies *R. p. praetor* and all mainland New Guinea populations belong to *R. p. coenorum*. Approximate sea levels of 50 m (dark grey) and 100 m (light grey) below modern mean sea level are indicated and represent the likely sea levels at 12 and 21 Ka BP respectively (Sathiamurthy and Voris, 2006; Voris, 2000). Points reflect location of specimens used in this study and colours indicate group classification identified using clustering analyses. (B) PCA of island (solid line) versus mainland (dashed line) tooth shape for *R. praetor*, with colours matching the results found from the morphological cluster analyses. The mean tooth shape of each group with additional landmarks to capture the curves of the cusps is plotted on the PCA in its group’s corresponding colour (see SI 1 and SI Fig. 1 for mean tooth shapes of analysed landmark configurations).

The genus *Rattus* has one of the most complex taxonomies among mammals, which is regularly revised (Musser and Carleton, 2005; Pagès et al., 2010; Robins et al., 2014; Timm et al., 2016). Members of this genus are morphologically homogenous despite numerous radiations (Schenk et al., 2013) and, therefore, is an excellent test system for methods to detect cryptic diversity. One third of *Rattus* species are found in New Guinea, Australia and neighbouring islands (Fig. 1) (Rowe et al., 2011). The lack of external morphological diversity of some species groups in this geographic region has demonstrably created difficulties in defining, delimiting and identifying species (Rowe et al., 2011; Taylor et al., 1982). In particular, *R. praetor* is an excellent candidate for investigating the presence of cryptic species because of its wide distribution across multiple habitats, and the earlier description of a range of proposed ecomorphs associated with different environments (Taylor et al., 1982). Distributed across New Guinea, the Bismarck Archipelago and the Solomon Islands, this species is a member of the Sahulian *Rattus* group and is divided into two subspecies: a New Guinea mainland form, *R. praetor coenorum* and an insular form *R. praetor praetor* spanning from the islands northeast of New Guinea to the Solomon islands (Rowe et al., 2011; Taylor et al., 1982). *R. praetor* appears to have had a wider distribution in antiquity, which included now extinct populations in Remote Oceania likely introduced by humans (White et al., 2000), but it is unclear which (if either) subspecies these extinct Remote Oceanic populations represent. Furthermore, the current subspecific division is also regarded as largely arbitrary, due to problems with full sampling across the distribution of *R. praetor* and lack of consistent/agreed morphological criteria (Taylor et al., 1982: p. 217). Morphological reanalyses of *R. praetor* is, therefore, required to better understand and re-appraise its ambiguous taxonomic status.

Identification of *R. praetor* is largely based on size, as it is the largest *Rattus* species of the Sahulian group (Robins et al., 2014; Rowe et al., 2011; Taylor et al., 1982). *R. praetor* is noted as being phenotypically highly variable, which has led to difficulties in its taxonomic assessment in previous studies (Robins et al., 2007; Taylor et al., 1982, p. 215). The high variability may have led to confusion with other Sahulian rats: *R. steini, R. mordax, R. jobiensis* and *R. novaeguineae*, and is a likely indicator of multiple cryptic species within *R. praetor* (Taylor et al., 1982). However, specific cryptic species divisions have so far not been suggested and morphological research has simply indicated ecomorphs generally associated with altitude (Taylor et al., 1982). More recent research has focused on genetic analyses for these species and the wider *Rattus* genus (Aplin et al., 2011; Pagès et al., 2010; Robins et al., 2008, 2014; Rowe et al., 2011). Some of these wider Sahulian *Rattus* genetic studies tentatively support earlier suggestions of the subdivision of *R. praetor* into a geographically distributed species complex (Robins et al., 2014), though without defining component species. Further, *R. praetor*’s position within the rapid Sahulian radiation is unclear and it appears to be closely related to (and possibly hybridising with) various other species such as *R. steini* (Robins et al., 2014). In light of the wide geographic distribution of *R. praetor* and reports of both geographic structure and ecomorphs (Robins et al., 2014; Taylor et al., 1982), it is highly likely there are discrete cryptic species that have yet to be identified.

Here we use geometric morphometrics as an exploratory tool to examine the dental morphology of *R. praetor* from across its range to determine subdivisions potentially attributable to separate species (in a similar fashion as studies in the Cypriot mouse [*Mus cypriacus*] Cucchi et al., 2006; and parasitic wasps [*Eubazus*] Villemant et al., 2007). Teeth are regularly recovered from archaeological and paleontological contexts and they provide a particularly useful element for identification of species and tracking past species’ distributions and evolution (Cucchi et al., 2014). Previous examination of *R. praetor* tooth shape have compared multiple linear measurements (e.g. White et al., 2000), but geometric morphometrics provides a much more powerful tool for quantifying shape, which cannot be adequately quantified using linear measurements (Adams et al., 2004). Applications of geometric morphometrics to the teeth of murids, especially *Mus*, have proven a powerful tool for inter- and intra-specific discrimination (Claude, 2013; Cucchi et al., 2006, Cucchi et al., 2011, Cucchi et al., 2013; Macholán, 2006; Valenzuela et al., 2009). In assessing the dental morphology of *R. praetor* populations from across New Guinea and neighbouring islands, we demonstrate the power of this morphometric approach and highlight how it should be used in conjunction with genetic studies in the future.

## Material and methods

The forty-eight specimens used in this study— and representing the species range (Fig. 1A)—were from the Bishop Museum, Hawaii (19); Naturalis, Leiden (20); and the Smithsonian Museum of Natural History, Washington D.C. (9). The museum labelling of the specimens noted thirteen unique locations (Table 1), but of varying degrees of precision (i.e. some locations represent a potential sampling area of only a few square kilometres, while others referred to whole regions of around a thousand square kilometres, see SI 2). Mandibular first molars of the specimens were photographed in the occlusal view using a Nikon E995 camera. All were from adults matched as far as possible to equivalent degrees of tooth wear. Environmental variables relating to rainfall and temperature derive from WorldClim (Hijmans et al., 2005). BioClim data was loaded to QGIS (2016) and geographic coordinates were used to extract the data.

**Table 1:** Sample information table. This table includes the unique locations from museum labelling, the approximate area that location represents and the number of specimens from that location in this study.

Landmass	Location	Approximate location area (km^2^)	Sample size
New Guinea	Bokondini	1000	4
New Guinea	Bulung River, Huongulf	1000	2
New Guinea	Prauwenbivak	10	1
New Guinea	Sibil Valley	2000	2
New Guinea	Teluk Etna	1400	11
New Guinea	West Sepik, Brugnowi	20	4
New Guinea	West Sepik, May River	40	2
Bougainville Island	Cape Torokina	30	9
Bougainville Island	Pokapa, SW of Tinputz	100	3
Bougainville Island	Tinputz	10	3
Bougainville Island	Mt. Karaea, Wakonvasike	unknown	1
Bougainville Island	Mutahi, SW of Tinputz	20	3
Bougainville Island	Mt. Balbi	20	3

**Fig. 2:**
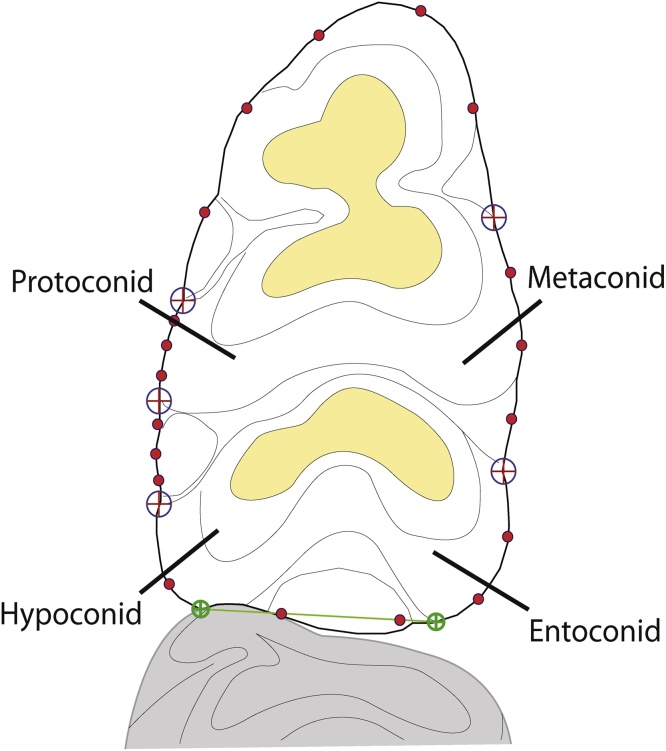
Schematic of a *Rattus* first left mandibular molar showing landmark protocol.

Landmarks and semi-landmarks were recorded from the photographs using tpsDig 2.12 (Rohlf, 2004) for geometric morphometric analyses. Five fixed Type 2 landmarks (according to the Bookstein, 1991 categorisation) were recorded and 19 sliding semi-landmarks were used to describe the outline of the teeth (adapted from Hulme-Beaman et al., 2018, see SI 1). The outline landmarks were recorded in five curves consisting of: 3, 5, 3, 3 and 5 sliding semilandmarks respectively (Fig. 2). An artificial straight line was taken on the posterior part of the tooth from the maximum posterior point of the hypoconid to the maximum posterior point of the entoconid to remove variation introduced from the second molar because in fossil and sub-fossil material the second molar may not be present (following Hulme-Beaman et al., 2018).

The landmark data were aligned using a generalised Procrustes analyses in R using the Geomorph package (Adams et al., 2017). Semi-landmarks were slid during this stage using the minimum Procrustes distance method (Sampson et al., 1996). Shape variables were extracted by carrying out a principal components analysis (PCA) on aligned configurations of landmarks. These shape variables were then used for further statistical analyses. The resulting principal components (PCs) were plotted, the distribution of shapes examined and the PCs used for further statistical analyses. Due to the high dimensionality of the data, it is generally considered appropriate to reduce the dimensionality to a lower number of principal components (Sheets et al., 2006). Dimensionality reduction was approached in different ways depending on the statistical procedure carried out (see below).

Differences in tooth morphology between island and mainland New Guinea were tested using a Procrustes ANOVA applied directly to the aligned landmark configurations (Klingenberg and McIntyre, 1998). The variance of mainland and island populations was calculated as the mean squared distance of each individual to the group mean and tested using an analysis of multivariate homogeneity of group dispersions—a multivariate analogue of Levene’s test, using the vegan package in R (Oksanen et al., 2017). To assess if geographic variation in shape correlated with climate, an RV test (10,000 iterations) was carried out on the superimposed configuration of shape coordinates and climatic variables (Heo and Gabriel, 1998).

A Gaussian mixture model cluster analysis was carried out on the principal components using the mclust package (Fraley et al., 2012) without *a priori* grouping. This approach produces varying results depending on the number of dimensions used. Therefore, dimensionality reduction was carried out in a stepwise manner, starting with principal component one and consecutively adding a principal component and returning the number of clusters found at each step. When > 2 principal components were used for the clustering analyses, we incorporated a clustering analysis with dimensional reduction following Scrucca (2010) to provide a summary plot of the underlying structure between multiple dimensions. This also indicates cluster boundaries and the position of specimens relative to those boundaries indicates levels of uncertainty.

The clusters found using the Gaussian mixture model were tested by permutation to see if each had a geographic component. A geographic distance matrix was also generated in R using the Imap package (Wallace, 2015). This calculated the geographic distance in kilometres between the geographic location of each specimen in the dataset. The geographic distance data were permuted 10,000 times for each cluster and the mean of each of those permutations was calculated. This was done by standard permutation, by randomly sampling the total dataset of distances between individual specimens to a sample size equal to the identified cluster sample size iteratively. The mean geographic distance between the individuals for each group was calculated at each iteration and a distribution of permuted mean distances was created. The mean of this permuted distribution of means was calculated. A one sample Wilcoxon test was used to test if the permuted mean was significantly greater than the distribution of real geographic distances, considering the permuted mean as μ. This considers that if the real distribution of distances is significantly less than the permuted mean, it can be considered that the geographic distribution of a group is non-random relative to the overall dataset. A one sample Wilcoxon test was used because the distances among specimens was not normally distributed due to distance data being bounded by zero (distance variables cannot be negative and in this case there were multiple specimens from the same or nearby locations).

Tooth size was quantified using the centroid size method, which was calculated for each individual as the square root of the sum of the squared distances between each landmark and the centroid of the specimen. Centroid size of island versus mainland *R. praetor* and groups identified from the Gaussian mixture model clustering analyses were tested using a Welch’s ANOVA in R. Levene’s test was used to test differences in centroid size variance.

## Results

PCA of tooth shape indicated the possible presence of multiple geographical forms because the Bougainville Island population separated from the New Guinea mainland population (Fig. 1B), with the differences statistically significant based on the Procrustes ANOVA (Df = 1, Sum Sq. = 0.046, Mean Sq. = 0.046, F = 24.9, *p* < 0.001). The variance in tooth shape values for the New Guinea mainland population was significantly greater than the island population based on the analysis of multivariate homogeneity of group dispersions (*p* < 0.001, mainland variance = 0.0012, island variance = 0.0007). The mean centroid sizes of the island and mainland populations were not found to be significantly different when tested using a Welsh’s ANOVA (Df = 1, Sum Sq. = 0.043, Mean Sq. = 0.0431, F = 0.071, *p *= 0.79). However, the island population exhibited significantly reduced variance compared with the mainland population (Levene’s test, Df = 1, F = 22.727, *p *< 0.001). None of the results of the RV test on environmental variables versus shape were significant (all *p *> 0.05 for the total dataset, New Guinea only and Island only, but see SI 3 for more details).

The stepwise approach to the Gaussian mixture model identified two groups using the first principal component (representing 55.53% of variance, see SI 4 for breakdown of principal component variance) and using the first three principal components (representing a cumulative variance of 79.41%). Three groups were identified using the first two principal components (representing a cumulative variance of 68.15%). A spherical equal volume model (EII) was found to be the best fit and identified the three groups using the first two principal components. The different dimensionalities (1 v 1–3 principal components) produced identical groupings. The two clusters identified comprised a predominantly western group and a predominantly eastern group, with mixed populations in the centre (See SI Fig. 2). For the analyses that found three clusters, the specimens of each identified cluster were highly geographically structured (Fig. 1) and Wilcoxon tests comparing the permuted mean distance versus the distribution of distances among specimens were all highly significant (*p* < 0.001 in each case). Cluster 1 was 80.77% composed of Bougainville Island specimens, cluster 2 was 76.92% composed of west New Guinea specimens and cluster 3 was 88.89% composed of central New Guinea specimens (Fig. 1). There were no differences between the mean centroid sizes for the clusters from a Welsh’s ANOVA (*p *> 0.05), nor were there differences in variance of the centroid sizes for the clusters (*p *> 0.05).

## Discussion

### Morphology of *Rattus praetor* in relation to geography and environmental factors

We found clear indications that the morphological variation of *R. praetor* across its distribution reflects a greater level of taxonomic division than previously recognised. Despite the genus *Rattus* and its component species often being described as morphologically homogenous (Rowe et al., 2011; Schenk et al., 2013), geometric morphometric analyses revealed quantifiable and interpretable signals in dental morphology. Three groups of *R. praetor* were observed from the dental shape data that have closely conforming geographical distributions: one Bougainville Island group, one central New Guinea group and one western New Guinea group. There are overlaps between some of these groups in central New Guinea, but it is not possible to identify the cause of these overlaps—i.e. due to recent human-mediated translocation, or similarity of morphology of intermediate forms or populations, or hybridisation. Furthermore, the geographic signal found here is greater than the difference in dental morphology between well established species in previous studies (i.e. Hulme-Beaman et al., 2018). To understand this further would require an in depth molecular analyses, incorporating nuclear markers (see below). Nevertheless, the clear grouping observed in these data indicate the current taxonomic divisions (subspecific or specific) are inadequate.

The higher level of morphological variance in New Guinea *R. praetor* relative to those from Bougainville Island conforms to the assessment made by Taylor et al. (1982) that New Guinea *R. praetor* are highly diverse. However, to fully understand this would require comparisons with other Sahulian *Rattus* species. The geographic structure in our data provides strong evidence for the presence of cryptic groups that likely stem from geographical barriers and local selective pressures across the highly biodiverse region of New Guinea with its variable mountainous and lowland terrain (Mittermeier et al., 1998), that should also be reflected in studies of other species.

Human-mediated introductions (White et al., 2000), the resulting founder events and subsequent adaptation to new niches could account for some of the observed shape variation. This would be particularly the case for Bougainville Island (and more widely the entire Bismarck Archipelago) as these regions have less faunal and floral diversity than New Guinea, so local adaptation to a different array of resources is possible. However, we found no evidence for ecomorphs as no correlations between climatic and shape variables were significant. Therefore, if the morphological differences between Bougainville Island and New Guinea populations are adaptive, there is no evidence they are influenced by climate; other factors, such as the influence of differences in biodiversity, are likely needed to explain regional shape diversity. We cannot speculate on functional differences between these dental morphologies, because we do not have dietary data. However, the poor evidence for ecomorphs from New Guinea *R. praetor* and recent evidence for phylogenetic signals from Mainland Southeast Asian *Rattus* dental morphology (Hulme-Beaman et al., 2018), would likely indicate that these differences in morphology may largely be driven by genetic drift. Furthermore, rapid divergence between populations of murid species is common where rugged terrain has likely reduced gene flow between populations (e.g. Madeiran *Mus musculus,* Britton-Davidian et al., 2000), as might be the case for vast regions of the mosaic of mountainous and lowland parts of New Guinea.

The geographic structure of the morphological dataset, showing no overall correlation with climatic factors, suggests that these morphological signals indicate population-wide differences. This does not discount the existence of ecomorphs, instead it indicates wider population differences are driving the observations made in this dataset. As a result, any morphological changes associated with ecological and climatic gradients would likely be masked by the overriding difference between populations. It is, therefore, possible that once a better understanding of the subspecific or specific divisions of *R. praetor* is established, ecomorphs will be identifiable for the individual species or subspecies, such as those altitudinal ecomorphs suggested by Taylor et al. (1982). Although the coverage of our sampling has not greatly improved on Taylor et al.’s (1982) work and future studies should expand sampling further, these results demonstrate that geometric morphometric analyses find sufficient and regular morphological differences between *R. praetor* populations and, as such, provide further evidence that the species should be taxonomically reconsidered.

### Relevance of the results to the genus *Rattus*

Our results bolster previous indications of phylogeographic structure from small scale sampling and genetic analysis (e.g. Robins et al., 2014). Given that this phylogeographic signal was tentatively interpreted as subdivision into cryptic species (Robins et al., 2014), our finding of morphological groups is of considerable interest. Our morphological findings are particularly important considering that *Rattus* are generally considered to be morphologically conserved. This group, therefore, merits urgent further investigation with increased sampling in the region of New Guinea that has in the past (and continues to be) under threat from lack of consistency in conservation (Whitfeld et al., 2014).

Genotypic data were not available from the same specimens as those examined here, so it was not possible to assess if the phylogeographic signal emerging from recent molecular studies (Robins et al., 2014) reflects our *R. praetor* morphological groups. However, a comparison between genetic and morphological datasets should be considered in the future. We did not examine the dental morphology of any other Sahulian *Rattus* species, and future morphological examination of other species is also needed. Mitochondrial DNA data on Sahulian *Rattus* have not been very helpful as a taxonomic tool for *R. praetor*, particularly due to problems in separating *R. praetor* and *R. steini*, as a consequence of hybridisation or incomplete mitochondrial lineage sorting (Robins et al., 2014). The extent of this hybridisation or incomplete lineage sorting in *R. praetor* is currently unknown (Robins et al., 2014). For direct comparison of morphological and genetic datasets in *R. praetor* and related species, both nuclear and full mitochondrial genomic data are required.

Given the mitochondrial results, it is possible that one or more of the groups we identified results from varying levels of admixture between closely related species, such as *R. steini* with *R. praetor*. It might be possible for future analyses to consider the extent of genetic admixture and then examine if this has a similar signal in dental morphology for that geographic region. To understand the likelihood of *R. praetor* representing a species complex or a diverse single species, these results need to be set in the wider context of morphological variation of all Sahulian *Rattus* species and multiple lines of evidence are required (Carstens et al., 2013). Geometric morphometrics has proven to be an efficient tool in exploring morphological variation where relatively little variation was previously thought to exist (e.g. Rowe et al., 2011).

### Future questions: conflicting archaeological evidence and necessity for identification

The extent of *R. praetor*’s natural range is not entirely clear, with archaeological reviews suggesting it may have been introduced to the Solomon Islands due to its apparent absence in early cave sites until 3–4000 BP (White et al., 2000). Our geometric morphometrics results indicate the *R. praetor* of Bougainville Island are morphologically highly divergent from those of mainland New Guinea, raising the question of how this morphological difference occurred. There is good archaeological evidence for *Rattus* remains from late Pleistocene and early Holocene deposits found at various sites across the Bismarck Archipelago and the Solomon Islands (Marshall and Allen, 1991). However, these findings are based on using relative size to identify the rodent remains, and measurements of the mandible and tooth alveoli; all large *Rattus* specimens were attributed to one of two species: either *R. praetor* or *R. mordax* (Marshall and Allen, 1991; White et al., 2000, 1991).

*R. mordax* have predominantly been found in the early contexts and those identified as *R. praetor* in the later ones (Marshall and Allen, 1991; White et al., 1991). However, this distribution of *R. mordax* is contrary to the range identified in earlier literature, which identifies it as exclusively having an eastern mainland and southeastern insular New Guinea distribution (Taylor et al., 1982). It is possible, with the changes in climate evident over the last 20,000 years, *R. mordax* may have changed in size, leading to misidentification when identification is primarily based on size. A review of these materials using similar geometric morphometrics approaches would provide greater insights into the taxonomic status of the possible *R. praetor* species complex and the island distribution of this species. It could also provide insights into ancient human activity and movement. In this region of the world, where DNA preservation is poor, geometric morphometrics provides an excellent additional methodology for re-examination in the likely absence of biomolecular data from ancient specimens.

## References

[bib0005] Adams D.C., Collyer M.L., Kaliontzopoulou A., Sherratt E. (2017). Geomorph: Software for Geometric Morphometric Analyses. R Package Version 3.0.5.

[bib0010] Adams D.C., Rohlf F.J., Slice D. (2004). Geometric morphometrics: ten years of progress following the “revolution”. Ital. J. Zool..

[bib0015] Aplin K.P., Suzuki H., Chinen A.A., Chesser R.T., ten Have J., Donnellan S.C., Austin J., Frost A., Gonzalez J.P., Herbreteau V., Catzeflis F., Soubrier J., Fang Y.-P., Robins J., Matisoo-Smith E., Bastos A.D.S., Maryanto I., Sinaga M.H., Denys C., Van Den Bussche R.A., Conroy C., Rowe K., Cooper A. (2011). Multiple geographic origins of commensalism and complex dispersal history of Black Rats. PLoS One.

[bib0020] Bickford D., Lohman D.J., Sodhi N.S., Ng P.K., Meier R., Winker K., Ingram K.K., Das I. (2007). Cryptic species as a window on diversity and conservation. Trends Ecol. Evol..

[bib0025] Bookstein F.L. (1991). Morphometric Tools for Landmark Data. Geometry and Biology.

[bib0030] Britton-Davidian J., Catalan J., da Graça Ramalhinho M., Ganem G., Auffray J.-C., Capela R., Biscoito M., Searle J.B., da Luz Mathias M. (2000). Rapid chromosomal evolution in island mice. Nature.

[bib0035] Carstens B., Pelletier T.A., Reid N.M., Statler J.D. (2013). How to fail at species delimitation. Mol. Ecol..

[bib0040] Claude J. (2013). Log-shape ratios, Procrustes superimposition, elliptic Fourier analysis: three worked examples in R. Hystrix. Ital. J. Mammal..

[bib0045] Cucchi T., Barnett R., Martínková N., Renaud S., Renvoisé E., Evin A., Sheridan A., Mainland I., Wickham-Jones C., Tougard C., Quéré J.P., Pascal M., Pascal M., Heckel G., O’Higgins P., Searle J.B., Dobney K.M. (2014). The changing pace of insular life: 5000 years of microevolution in the Orkney vole (*Microtus arvalis orcadensis*). Evolution.

[bib0050] Cucchi T., Hulme-Beaman A., Yuan J., Dobney K. (2011). Early Neolithic pig domestication at Jiahu, Henan Province, China: clues from molar shape analyses using geometric morphometric approaches. J. Archaeol. Sci..

[bib0055] Cucchi T., Kovács Z.E., Berthon R., Orth A., Bonhomme F., Evin A., Siahsarvie R., Darvish J., Bakhshaliyev V., Marro C. (2013). On the trail of Neolithic mice and men towards Transcaucasia: zooarchaeological clues from Nakhchivan (Azerbaijan). Biol. J. Linn. Soc..

[bib0060] Cucchi T., Orth A., Auffray J.-C., Renaud S., Fabre L., Catalan J., Hadjisterkotis E., Bonhomme F., Vigne J.-D. (2006). A new endemic species of the subgenus *Mus* (Rodentia, Mammalia) on the Island of Cyprus. Zootaxa.

[bib0065] Fišer C., Robinson C.T., Malard F. (2018). Cryptic species as a window into the paradigm shift of the species concept. Mol. Ecol..

[bib0070] Fraley C., Raftery A.E., Murphy T.B., Scrucca L. (2012). mclust Version 4 for R: Normal Mixture Modeling for Model-Based Clustering, Classification, and Density Estimation.

[bib0075] Heo M., Gabriel K.R. (1998). A permutation test of association between configurations by means of the RV coefficient. Commun. Stat.-Simul. Comput..

[bib0080] Hijmans R.J., Cameron S.E., Parra J.L., Jones P.G., Jarvis A. (2005). Very high resolution interpolated climate surfaces for global land areas. Int. J. Climatol..

[bib0085] Hulme-Beaman A., Claude J., Chaval Y., Evin A., Morand S., Vigne J.-D., Dobney K., Cucchi T. (2018). Dental shape variation and phylogenetic signal in the Rattini tribe species of Mainland Southeast Asia. J. Mamm. Evol..

[bib0090] Klingenberg C.P., McIntyre G.S. (1998). Geometric morphometrics of developmental instability: analyzing patterns of fluctuating asymmetry with Procrustes methods. Evolution.

[bib0095] Macholán M. (2006). A geometric morphometric analysis of the shape of the first upper molar in mice of the genus *Mus* (Muridae, Rodentia). J. Zool..

[bib0100] Marshall B., Allen J. (1991). Excavations at Panakiwuk Cave, New Ireland. Occas. Pap. Prehist. Rep. Lapita Homal. Proj..

[bib0105] Michaux J., Reyes A., Catzeflis F. (2001). Evolutionary history of the most speciose mammals: molecular phylogeny of muroid rodents. Mol. Biol. Evol..

[bib0110] Mittermeier R.A., Myers N., Thomsen J.B., da Fonseca G.A.B., Olivieri S. (1998). Biodiversity hotspots and major tropical wilderness areas: approaches to setting conservation priorities. Conserv. Biol..

[bib0115] Musser G.G., Carleton M.D., Wilson D.E., Reeder D.M. (2005). Superfamily Muroidea. Mammal Species of the World: A Taxonomic and Geographic Reference.

[bib0120] Oksanen J., Blanchet F.G., Friendly M., Kindt R., Legendre P., McGlinn D., Minchin P.R., O’Hara R.B., Simpson G.L., Solymos P., Stevens M.H.H., Szoecs E., Wagner H. (2017). Vegan: Community Ecology Package 2.4-2.

[bib0125] Pagès M., Bazin E., Galan M., Chaval Y., Claude J., Herbreteau V., Michaux J., Piry S., Morand S., Cosson J.-F. (2013). Cytonuclear discordance among Southeast Asian black rats (*Rattus rattus* complex). Mol. Ecol..

[bib0130] Pagès M., Chaval Y., Herbreteau V., Waengsothorn S., Cosson J.-F., Hugot J.-P., Morand S., Michaux J. (2010). Revisiting the taxonomy of the Rattini tribe: a phylogeny-based delimitation of species boundaries. BMC Evol. Biol..

[bib0135] Paupério J., Herman J.S., Melo-Ferreira J., Jaarola M., Alves P.C., Searle J.B. (2012). Cryptic speciation in the field vole: a multilocus approach confirms three highly divergent lineages in Eurasia. Mol. Ecol..

[bib0140] QGIS D.T. (2016). QGIS Geographic Information System. Open Source Geospatial Foundation Project.

[bib0145] Robins J.H., Hingston M., Matisoo-Smith E., Ross H.A. (2007). Identifying *Rattus* species using mitochondrial DNA. Mol. Ecol. Notes.

[bib0150] Robins J.H., McLenachan P.A., Phillips M.J., Craig L., Ross H.A., Matisoo-Smith E. (2008). Dating of divergences within the *Rattus* genus phylogeny using whole mitochondrial genomes. Mol. Phylogenet. Evol..

[bib0155] Robins J.H., Tintinger V., Aplin K.P., Hingston M., Matisoo-Smith E., Penny D., Lavery S.D. (2014). Phylogenetic species identification in *Rattus* highlights rapid radiation and morphological similarity of New Guinean species. PLoS One.

[bib0160] Rohlf F.J. (2004). TpsDig 1.40-Thin Plate Spline Digitizer.

[bib0165] Rowe K.C., Aplin K.P., Baverstock P.R., Moritz C. (2011). Recent and rapid speciation with limited morphological disparity in the genus *Rattus*. Syst. Biol..

[bib0170] Sampson P.D., Bookstein F.L., Sheehan F.H., Bolson E.L., Marcus L.F., Corti M., Loy A., Naylor G.J.P., Slice D.E. (1996). Eigenshape analysis of left ventricular outlines from contrast ventriculograms.

[bib0175] Sathiamurthy E., Voris H.K. (2006). Maps of Holocene sea level transgression and submerged lakes on the Sunda Shelf. Nat. Hist..

[bib0180] Schenk J.J., Rowe K.C., Steppan S.J. (2013). Ecological opportunity and incumbency in the diversification of repeated continental colonizations by muroid rodents. Syst. Biol..

[bib0185] Scrucca L. (2010). Dimension reduction for model-based clustering. Stat. Comput..

[bib0190] Sheets H.D., Covino K.M., Panasiewicz J.M., Morris S.R. (2006). Comparison of geometric morphometric outline methods in the discrimination of age-related differences in feather shape. Front. Zool..

[bib0195] Taylor J.M., Calaby J.H., Van Deusen H.M. (1982). A revision of the genus *Rattus* (Rodentia, Muridae) in the New Guinean region. Bull. Am. Mus. Nat. Hist..

[bib0200] Timm R.M., Weijola V., Aplin K.P., Donnellan S.C., Flannery T.F., Thomson V., Pine R.H. (2016). A new species of *Rattus* (Rodentia: Muridae) from Manus Island, Papua New Guinea. J. Mammal..

[bib0205] Valenzuela S., Poitevin F., Cornette R., Bournery A., Nadal J., Vigne J.-D. (2009). Evolving ecosystems: ecological data from an Iron Age small mammal accumulation at Alorda Park (Catalonia, Spain). J. Archaeol. Sci..

[bib0210] Villemant C., Simbolotti G., Kenis M. (2007). Discrimination of *Eubazus* (Hymenoptera, Braconidae) sibling species using geometric morphometrics analysis of wing venation. Syst. Entomol..

[bib0215] Voris H.K. (2000). Maps of Pleistocene sea levels in Southeast Asia: shorelines, river systems and time durations. J. Biogeogr..

[bib0220] Wallace J.R. (2015). “Imap” Package 1.32.

[bib0225] White J.P., Clark G., Bedford S. (2000). Distribution, present and past, of *Rattus praeto*r in the Pacific and its implications. Pac. Sci..

[bib0230] White J.P., Flannery T.F., O’Brien R., Hancock R.V., Pavlish L. (1991). The Balof shelters, New Ireland. Occas. Pap. Prehist. Rep. Lapita Homal. Proj..

[bib0235] Whitfeld T.J.S., Lasky J.R., Damas K., Sosanika G., Molem K., Montgomery R.A. (2014). Species richness, forest structure, and functional diversity during succession in the New Guinea Lowlands. Biotropica.

