# Supplementary material for: Exploring Rattus praetor (Rodentia, Muridae) as a possible species complex using geometric morphometrics on dental morphology
Source: Mamm Biol. 2018 Sep;92:62–7. doi: 10.1016/j.mambio.2018.04.002 (PMC6067089; doi:10.1016/j.mambio.2018.04.002)
Supplement: Supplementary file 1 [file mmc1.docx]

**Supplementary Information**

**SI 1. Semilandmarks and mean shapes of *Rattus praetor***

For the purposes of visualising the different tooth shapes for the three morphological clusters identified we provided the mean shapes of the full set of geometric morphometric landmarks (after Hulme-Beaman et al., 2018). All analyses for this paper were carried out on both this full set of landmarks and also the reduced set of landmarks as described in the Material and methods section of the main text. The full set had previously been shown appropriate to distinguish species where subtle differences in outline were diagnostically important, e.g. particularly where the appearance, disappearance and size of microcusps was diagnostic (Musser, 1972; Musser and Heaney, 1985; Musser and Holden, 1991; Pimsai et al., 2014). However, for *R. praetor* we found it was a more general overall shape configuration of the major six cusps that drove the shape differences between morphological groups. Therefore, it was possible and desirable to reduce the semilandmarks down to a smaller subset. Both the full set of landmarks and the reduced set produced near identical results. SI Figure 1 shows the mean shapes for the reduced set of landmarks.

Musser, G.G., 1972. Identities of Taxa Associated with Rattus rattus (Rodentia, Muridae) of Sumba Island, Indonesia. J. Mammal. 53, 861–865.

Musser, G.G., Heaney, L.R., 1985. Philippine Rattus: A New species from the Sulu Archipelago. Am. Museum Novit.

Musser, G.G., Holden, M.E., 1991. Sulawesi Rodents (Muridae: Murinae): Morphological and Geographical Boundaries of Species in the Rattus hoffmanni Group and a New Species from Pulau Peleng. Bull. Am. Museum Nat. Hist. 206, 322–413.

Pimsai, U., Pearch, M.J., Satasook, C., Bumrungsri, S., Bates, P.J.J., 2014. Murine rodents (Rodentia: Murinae) of the Myanmar-Thai-Malaysian peninsula and Singapore: taxonomy, distribution , ecology , conservation status , and illustrated identification keys. Bonn Zool. Bull. 63, 15–114.

**SI Figure 1.** Mean shapes of reduced semilandmark configuration. The top three mean shapes labelled 1, 2 and 3 are the mean shapes of each of the three shapes identified through clustering analyses. The lower two mean shapes are the shapes for the Bougainville Island and mainland New Guinea forms of *Rattus praetor*.

|  |  |  |
| --- | --- | --- |
|  |  |  |

**SI 2. Sampling locations of *Rattus praetor***

The level of sampling location information for *R. praetor* museum specimens was highly variable, with some locations representing ~10km^2^ and others >1000km^2^. To assess if there was particularly close clustering of specimens from the same recorded location we plotted the unique locations with the same points and convex hulls where multiple specimens were present. We plotted these on top of coloured points representing New Guinea (blue triangles) and Bougainville Island (orange circles) to help visually assess the similarity of specimens from the same uniquely labelled museum location (SI Figure 2). Visual inspection would suggest that specimens from the same labelled sampling location do not necessarily represent tighter groups than the Gaussian based clustering method we used on dental morphology to identified groups here.

**SI Figure 2.** *Rattus praetor* ecomorphs and morphology versus climate two-block partial least squares analyses. Bougainville Island specimens are marked by orange circles and mainland New Guinea specimens are marked by blue triangles. Different sampling locations are marked with different internal points with convex hulls joining multiple specimens from the same labelled sampling location.

**SI 3. Ecomorphs of *Rattus praetor* and two-block partial least squares of climate versus tooth shape variables**

Depending on the data we put into these analyses (full shape coordinates or shape principal components and raw climatic data or climate principal components) we got slightly different results for the RV test and for two block partial least squares analyses (Rohlf and Corti, 2000; carried out in the R package Geomorph, Adams et al., 2017). However, consistently the correlation was extremely low and only occasionally close to the significance limit of 0.05. The only regularly significant signal at the 0.05 limit was when the dataset was subdivided into New Guinea and Bougainville Island. When subdivided climate v shape two-block partial least square results from New Guinea were significant (*p* < 0.05) there was a low level of covariance explained (11–5%). Significant results were only achieved when principal components of shape and climate were used (9–11% of covariance). This may indicate that individual shape variation in the full dataset of Procrustes coordinates is greater than the signal from climate and therefore when full shape is used (instead of an abstract projection in PCA) the low level of climate signal is lost. Future analyses with larger sample sizes and better taxonomic divisions of *R. praetor* may find significant shape-climate correlations for each taxon (whatever that may be: i.e. population, subspecies or species). However, with the current sampling the wider geographic shape differences mask any climatic variation. Therefore, this result was not robust enough to confidently interpret and we currently consider there to be no good evidence from dental morphology for ecomorphs.

**References:**

Adams, D.C., Collyer, M.L., Kaliontzopoulou, A., Sherratt, E., 2017. Geomorph: Software for geometric morphometric analyses. R package version 3.0.5. <https://cran.r-project.org/package=geomorph>.

Rohlf, F.J., Corti, M., 2000. Use of two-block partial least-squares to study covariation in shape. Syst. Biol. 49, 740–753.

**SI 4. Summary of principal component variance of *Rattus praetor* tooth morphology**

**Table SI 1.** A table of the variance associated with each principal component. The standard deviation (SD), the proportion of variance (% Variance) and the cumulative proportion of variance (Cumulative %) for each principal component is listed below

|  | PC1 | PC2 | PC3 | PC4 | PC5 | PC6 | PC7 | PC8 | PC9 | PC10 |
| --- | --- | --- | --- | --- | --- | --- | --- | --- | --- | --- |
| SD | 0.0392 | 0.0187 | 0.0176 | 0.0122 | 0.0108 | 0.0094 | 0.007 | 0.0067 | 0.0052 | 0.0045 |
| % Variance | 0.5553 | 0.1263 | 0.1126 | 0.0536 | 0.0421 | 0.0318 | 0.0177 | 0.0163 | 0.0098 | 0.0073 |
| Cumulative % | 0.5553 | 0.6816 | 0.7941 | 0.8478 | 0.8899 | 0.9217 | 0.9393 | 0.9557 | 0.9655 | 0.9728 |
|  | PC11 | PC12 | PC13 | PC14 | PC15 | PC16 | PC17 | PC18 | PC19 | PC20 |
| SD | 0.004 | 0.0036 | 0.0032 | 0.0028 | 0.0025 | 0.0022 | 0.0019 | 0.0018 | 0.0016 | 0.0012 |
| % Variance | 0.0058 | 0.0047 | 0.0036 | 0.0029 | 0.0023 | 0.0017 | 0.0013 | 0.0011 | 9.00E-04 | 5.00E-04 |
| Cumulative % | 0.9786 | 0.9833 | 0.9869 | 0.9898 | 0.9921 | 0.9938 | 0.9951 | 0.9962 | 0.9971 | 0.9976 |
|  | PC21 | PC22 | PC23 | PC24 | PC25 | PC26 | PC27 | PC28 | PC29 | PC30 |
| SD | 0.0011 | 0.0011 | 9.00E-04 | 9.00E-04 | 7.00E-04 | 6.00E-04 | 5.00E-04 | 5.00E-04 | 5.00E-04 | 4.00E-04 |
| % Variance | 5.00E-04 | 4.00E-04 | 3.00E-04 | 3.00E-04 | 2.00E-04 | 1.00E-04 | 1.00E-04 | 1.00E-04 | 1.00E-04 | 1.00E-04 |
| Cumulative % | 0.9981 | 0.9985 | 0.9988 | 0.9991 | 0.9993 | 0.9994 | 0.9995 | 0.9996 | 0.9997 | 0.9998 |
|  | PC31 | PC32 | PC33 | PC34 | PC35 | PC36 | PC37 | PC38 | PC39 | PC40 |
| SD | 4.00E-04 | 3.00E-04 | 3.00E-04 | 3.00E-04 | 2.00E-04 | 2.00E-04 | 2.00E-04 | 2.00E-04 | 1.00E-04 | 1.00E-04 |
| % Variance | 0 | 0 | 0 | 0 | 0 | 0 | 0 | 0 | 0 | 0 |
| Cumulative % | 0.9998 | 0.9999 | 0.9999 | 0.9999 | 1 | 1 | 1 | 1 | 1 | 1 |
|  | PC41 | PC42 | PC43 | PC44 | PC45 | PC46 | PC47 | PC48 |  |  |
| SD | 1.00E-04 | 1.00E-04 | 1.00E-04 | 0 | 0 | 0 | 0 | 0 |  |  |
| % Variance | 0 | 0 | 0 | 0 | 0 | 0 | 0 | 0 |  |  |
| Cumulative % | 1 | 1 | 1 | 1 | 1 | 1 | 1 | 1 |  |  |
